# Impact of an innovative financing and payment model on tuberculosis patients’ financial burden: is tuberculosis care more affordable for the poor?

**DOI:** 10.1186/s40249-019-0532-x

**Published:** 2019-03-24

**Authors:** Wei-Xi Jiang, Qian Long, Henry Lucas, Di Dong, Jia-Ying Chen, Li Xiang, Qiang Li, Fei Huang, Hong Wang, Chris Elbers, Frank Cobelens, Sheng-Lan Tang

**Affiliations:** 1 Global Health Research Center, Duke Kunshan University, Kunshan, 215316 Jiangsu China; 2 Institute of Development Studies, University of Sussex, Brighton, BN1 9RE UK; 3 School of Policy & Management, Nanjing Medical University, Nanjing, 211166 Jiangsu China; 4 Tongji Medical College of Huazhong University of Science and Technology, Wuhan, 430030 Hubei China; 5 School of Public Health, Xi’an Jiaotong University, Xi’an, 710061 Shaanxi China; 6 National Center for Tuberculosis Control and Prevention, China CDC, Beijing, 102206 China; 7 Bill & Melinda Gates Foundation, Seattle, WA USA; 8 Faculty of Economics and Business Administration, Vrije Universiteit Amsterdam, Amsterdam, 1081 HV the Netherlands; 9 The Amsterdam Institute for Global Health and Development, Amsterdam, 1105 BP the Netherlands; 10 Duke Global Health Institute, Duke University, Durham, NC 27710 USA

**Keywords:** Health insurance, Tuberculosis, Financing and payment model, Case-based payment

## Abstract

**Background:**

In response to the high financial burden of health services facing tuberculosis (TB) patients in China, the China-Gates TB project, Phase II, has implemented a new financing and payment model as an important component of the overall project in three cities in eastern, central and western China. The model focuses on increasing the reimbursement rate for TB patients and reforming provider payment methods by replacing fee-for-service with a case-based payment approach. This study investigated changes in out-of-pocket (OOP) health expenditure and the financial burden on TB patients before and after the interventions, with a focus on potential differential impacts on patients from different income groups.

**Methods:**

Three sample counties in each of the three prefectures: Zhenjiang, Yichang and Hanzhong were chosen as study sites. TB patients who started and completed treatment before, and during the intervention period, were randomly sampled and surveyed at the baseline in 2013 and final evaluation in 2015 respectively. OOP health expenditure and percentage of patients incurring catastrophic health expenditure (CHE) were calculated for different income groups. OLS regression and logit regression were conducted to explore the intervention’s impacts on patient OOP health expenditure and financial burden after adjusting for other covariates. Key-informant interviews and focus group discussions were conducted to understand the reasons for any observed changes.

**Results:**

Data from 738 (baseline) and 735 (evaluation) patients were available for analysis. Patient mean OOP health expenditure increased from RMB 3576 to RMB 5791, and the percentage of patients incurring CHE also increased after intervention. The percentage increase in OOP health expenditure and the likelihood of incurring CHE were significantly lower for patients from the highest income group as compared to the lowest. Qualitative findings indicated that increased use of health services not covered by the standard package of the model was likely to have caused the increase in financial burden.

**Conclusions:**

The implementation of the new financing and payment model did not protect patients, especially those from the lowest income group, from financial difficulty, due partly to their increased use of health service. More financial resources should be mobilized to increase financial protection, particularly for poor patients, while cost containment strategies need to be developed and effectively implemented to improve the effective coverage of essential healthcare in China.

**Electronic supplementary material:**

The online version of this article (10.1186/s40249-019-0532-x) contains supplementary material, which is available to authorized users.

## Multilingual abstracts

Please see Additional file 1 for translations of the abstract into the five official working languages of the United Nations

## Background

China is among the countries with the highest burden of tuberculosis (TB). According to the WHO Global Tuberculosis Report 2017, China ranked 3rd in terms of incident TB cases and 2nd on multi-drug resistant TB (MDR-TB) [1]. In order to achieve the health-related Sustainable Development Goal (SDG) proposed by the United Nations and end the TB epidemic by 2030, standard medical treatment needs to be made accessible and affordable for all TB patients [2].

Currently the TB service delivery system in China has been undergoing a transformation whereby responsibility for provision of clinical services is being gradually shifted from TB dispensaries to designated hospitals, typically general hospitals or infectious disease hospitals, in most of the provinces [3]. This transformation has had considerable cost implications, as studies have shown that, while quality of care has often been improved, income-pursuing behaviors, such as over-prescription and unnecessary hospitalization have driven up the treatment cost of TB [4–7]. Previous studies also show that although over 95% of the Chinese population is covered by the three public health insurance systems – Urban Employee Basic Medical Insurance (UEBMI), Urban Resident Basic Medical Insurance (URBMI) and New Cooperative Medical Schemes (NCMS) [8] – TB patients bear a high economic burden for medical treatment, especially low-income and rural patients [6, 9–12]. Financial factors are also frequently mentioned as a major reason for non-adherence to treatment, suspended treatment and eventual non-cure [13–17]. As a majority of TB patients live in poor households [18, 19], it seems likely that the financial protection provided by the current health insurance schemes is far from sufficient.

In response to the financial difficulties faced by patients, the China-Gates TB project, Phase II has implemented a new financing and payment model as one component of a set of interventions that aims at improving TB control in China. Before project implementation, studies from the baseline survey have found that the medical cost was relatively high in the project area [4]. The effect of NCMS on reducing catastrophic health expenditure (CHE) for TB patients was very limited [9], and the percentage of poor patients who incurred CHE was much higher than the rich [20]. The new model focuses on improving the reimbursement rate for TB patients and introducing hospital payment reforms, moving from a fee-for-service to a case-based payment approach. A treatment guideline was also launched with a standard service package, the cost of this being covered under the new model. Transportation and subsistence allowances are also provided to patients who adhere to treatment. The other components such as establishing a comprehensive TB control model, are documented elsewhere [12].

While increasing the reimbursement rate appears to have obvious potential benefits for patients, the implications of the case-based payment approach are not clear. By setting a fixed payment rate for each case, case-based payment aims to reduce non-necessary medical services by altering provider incentives. Diagnostic-related group (DRG) case-based payment mechanisms, have been implemented in numerous developed and developing countries, and in some cases have demonstrated effectiveness in cost containment [21–23]. Previous studies in China have found mixed results regarding the impact of case-based payments in terms of reducing medical costs for different types of disease, but little evidence of reduced overall inpatient expenditure [24–29]. In addition, it has been found that the impact may be compromised by problems that have arisen in project implementation, for example with hospitals excluding patients whose treatment cost is higher than the payment limit [25, 29]. For TB specifically, at least one study suggests that a case-based payment approach with no co-payment for patients may be feasible and beneficial under the NCMS [30]. However, there is a lack of evidence as to the effects in terms of cost control and patient financial burden of adopting a model combining increased reimbursement rates and case-based payment. In particular, it remains unknown whether this approach could protect the most economically vulnerable groups from financial difficulty.

At the end of the second phase of the China-Gates TB project, a team led by Duke Global Health Institute carried out an evaluation of the effects of the interventions on equity in access to/use of TB services, and on changes in financial protection for TB patients. This article aims to investigate changes in the out-of-pocket (OOP) health expenditure and financial burden of TB patients before and after the implementation of the new model, while a companion paper by Dong et al. which is published in the following issue considers changes in service utilization. It also examines the equity implications of the new model for patients from different socio-economic groups, especially those living in poverty.

## Methods

**Fig. 1:**
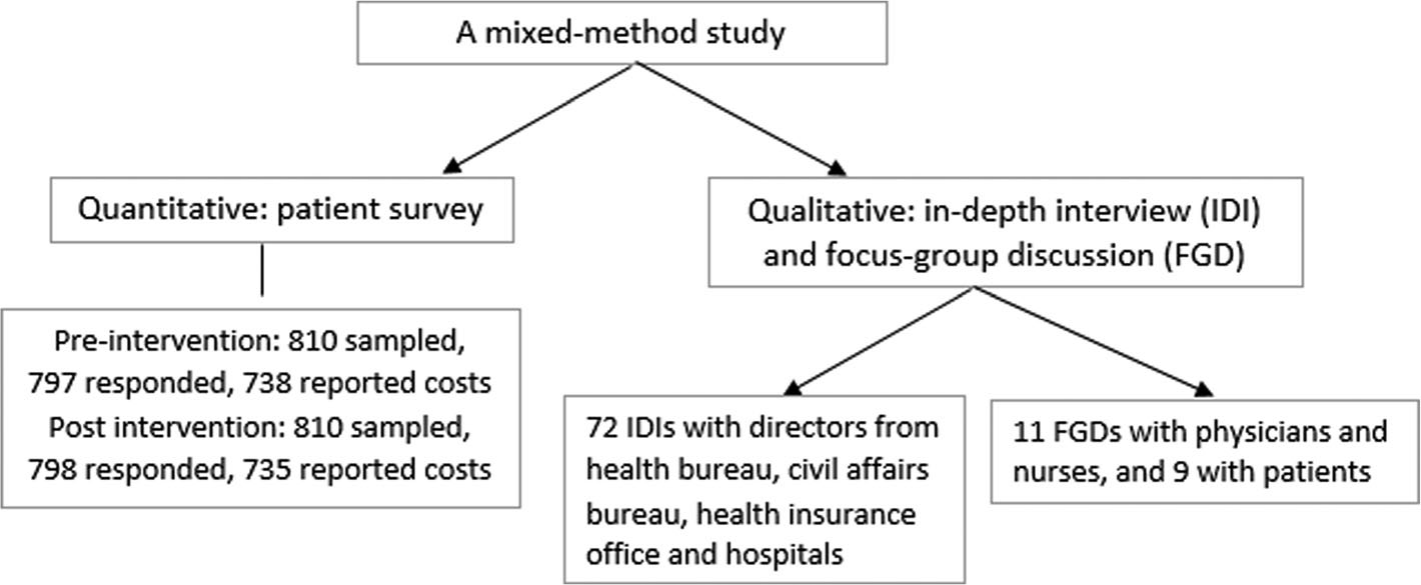
Flow chart of the study design

The study was conducted in Zhenjiang, Yichang and Hanzhong, three prefectures located in eastern, central and western China respectively. In each prefecture, three sample counties at different levels of economic development were selected, one economically disadvantaged and located in a remote area to ensure that we included a substantial number of patients living in poverty. A mixed-method approach, including a patient questionnaire survey and qualitative interviews, was used to investigate patients’ financial burden due to TB treatment. The study design is shown in Fig. 1.

### Data collection

#### Patient survey

The intervention period was from April 2014 to March 2015. The baseline survey was conducted in 2013 and the final evaluation late in 2015, with 90 patients randomly sampled from the list of registered TB patients in each county for both surveys. The inclusion criterion for the baseline required that patients had completed treatment before the survey, and for the final evaluation that patients started and completed treatment during the intervention period. Face-to-face interviews were conducted by trained investigators using a structured survey questionnaire which collected basic demographic information and data on socio-economic status, treatment procedures and expenditures. All questionnaires were checked on site for completeness and internal logic, and they were captured for analysis using a double-entry procedure in EpiData (http://www.epidata.dk/).

#### Qualitative interviews

Qualitative data were gathered during the evaluation of the project and used to gain an understanding of stakeholders’ perceptions of the effects of the new payment model on financial protection for TB patients. Semi-structured, in-depth interviews were conducted with participants responsible for the development and implementation of local policies, including officers of the local health bureau and civil affair bureau, health insurance managers and hospital managers at city-level and county-level, a total of 72 interviews across all study sites. Focus group discussions (FGD) were also conducted among care-givers (physicians and nurses, *n* = 11 in total) and TB patients who completed the 6-month first-line treatment during the intervention period (*n* = 9 in total), with each group consisting of 5–6 participants. Gender, household income, type of health insurance coverage and distance from downtown were considered to ensure diversity of participants in the patient FGDs. The topic guides were developed and piloted before formal interview. All qualitative interviews were conducted by scholars with qualitative study experience. All interviews were conducted in a private room, and were recorded after obtaining the oral consent from the interviewees.

### Data analysis

#### Quantitative

Out-of-pocket (OOP) health expenditure, calculated by adding up direct health expenditures for all outpatient and inpatient services, and the percentage of patients incurring CHE, defined as OOP health expenditure over 10% of annual household income or 40% of annual non-food expenditure [20], were used to evaluate patients’ treatment expenses and financial burden before and after the intervention. Chi-square tests were conducted to examine if there were significant differences between the baseline and final patient samples in terms of demographic and socio-economic characteristics including age, gender, insurance cover and household income.

In order to explore the new model’s potential differential impact on patients from different income groups, especially the effect on the poorest patients that are most susceptible to financial difficulty, patients were categorized, based on the per capita income of their household, as extremely poor (below the 1.9 USD per capita per day defined by the World Bank, which equated to 4369 RMB per capita per year), moderately poor (1.9–5.5 USD per capita per day or 4369–12 647 RMB per capita per year, defined by the World Bank as the poverty line for middle and high income countries) and non-poor (≥ 5.5 USD per capita per day or 12 647 RMB per capita per year) [31]. *T*-tests were used to determine if there were significant differences in OOP health expenditure before and after the intervention for all patients and for selected subgroups. Linear regression was used to model the effect of the intervention on the natural logarithm of OOP, and logistic regression to estimate the effect on the percentage of patients incurring CHE. The regression models were estimated both for all patients and for patients from different income groups, in both cases using other demographic and socio-economic factors as explanatory variables and controlling for county fixed effects. Crude and standardized (adjusting for age, gender and patient category) concentration indices for OOP health expenditure, were calculated to assess the degree of equity across income groups in the financing of TB treatment before and after the intervention [32]. The quantitative data were analyzed using STATA 13.1(StataCorp, Texas, USA).

#### Qualitative

The qualitative data were analyzed using the thematic analysis approach [33]. The analysis framework was developed based on a topic guide and emerging themes from the transcripts, and was refined during the coding process. All qualitative data were coded, sorted and classified according to the framework, and discussed across the research team till reaching consensus. The trustworthiness of data was enhanced by triangulating findings from different respondents and methods. We used the original Chinese texts for analysis, and translated the quotations into English. The analysis was conducted using NVIVO 9.0 (QSR International, Melbourne, Australia).

## Results

### Characteristics of the patients surveyed

A total of 797 and 798 patients agreed to participate in the baseline and evaluation surveys respectively, and 738 (baseline) and 735 (evaluation) reported their out-of-pocket health expenditure and were included in the analysis. Table 1 indicates that there were no significant differences between the baseline and final samples with respect to gender, age, employment, education and household income. Most were male and new patients. Around 50% were aged over 60, and 85% had never completed high school. Half were not employed at the time of the survey. Some 1/3 lived in households below the poverty line and 24% were above the non-poor threshold. Over 80% were covered by the NCMS, as most lived in rural area. Overall, most are likely to come from the low socio-economic group, as reflected by their education and income status.

**Table 1:** Characteristics of patient samples before and after intervention (%)

	Before intervention (*n* = 738)	After intervention (*n* = 735)	*P*-value^1^
Gender			
Male	555 (75.2%)	532 (72.4%)	0.218
Age			
< 30	45 (6.1%)	58 (7.9%)	0.065
30–59	352 (47.7%)	309 (42.0%)	
≥ 60	341 (46.2%)	468 (50.1%)	
Marital status			
Married	595 (80.6%)	575 (78.3%)	0.278
Patient category			
new patient	594 (81.4%)	602 (81.9%)	0.791
Education level			
None	140 (19.0%)	166 (22.7%)	0.119
Primary school	242 (32.8%)	247 (33.7%)	
Secondary school	262 (33.5%)	221 (31.1%)	
≥ high school	94 (12.7%)	99 (12.5%)	
Insurance type			
UEBMI	45 (6.1%)	69 (9.4%)	
resident insurance	21 (2.8%)	45 (5.9%)	
NCMS	648 (87.8%)	598 (81.6%)	0.000
other insurance	16 (2.2%)	5 (0.7%)	
no insurance	8 (1.1%)	18 (2.5%)	
Employment			
Employed	403 (54.6%)	369 (50.2%)	0.091^2^
Unemployed	40 (5.4%)	55 (7.5%)	
Retired	252 (34.2%)	86 (11.7%)	
lost ability	35 (4.7%)	91 (12.4%)	
other	8 (1.1%)	134 (18.2%)	
Income group^a^			
< 4369 RMB	243 (33.2%)	240 (33.5%)	0.970
4369–12 647 RMB	314 (42.9%)	303 (42.3%)	
> 12 647 RMB	175 (23.9%)	174 (23.2%)	

*UEBMI* Urban Employee Basic Medical Insurance, *NCMS* New Cooperative Medical Schemes

^1^ *P*-value for chi-square test. ^2^ *P*-value for comparison employed vs unemployed

^a^The numbers in the three categories do not add up to total due to missing data

### Out-of-pocket health expenditure

The mean OOP health expenditure was RMB 3576 (median 1752) before and RMB 5791 (median 2700) after the implementation of the new financing model (Table 2). The most significant increase (*P* <  0.001) was for the poorest, whose mean OOP expenditure more than doubled, while the moderately poor experienced an increase of almost 70%. Only for patients in the highest income group was the percentage increase relatively limited and not statistically significant.

**Table 2:** Out-of-pocket health expenditure before and after intervention by income group (RMB)

Income group	Before intervention		After intervention		% change		*P*-value
	Mean	Median	Mean	Median	Mean	Median	(Means)
< 4369 RMB	2876	1428	5961	2700	107.3	89.1	< 0.001
4369–12 647 RMB	3656	1752	6169	2780	68.7	58.7	0.002
> 12 647 RMB	4409	2400	4972	2570	12.8	7.1	0.4
Overall	3576	1752	5791	2700	61.9	54.1	< 0.001

**Fig. 2:**
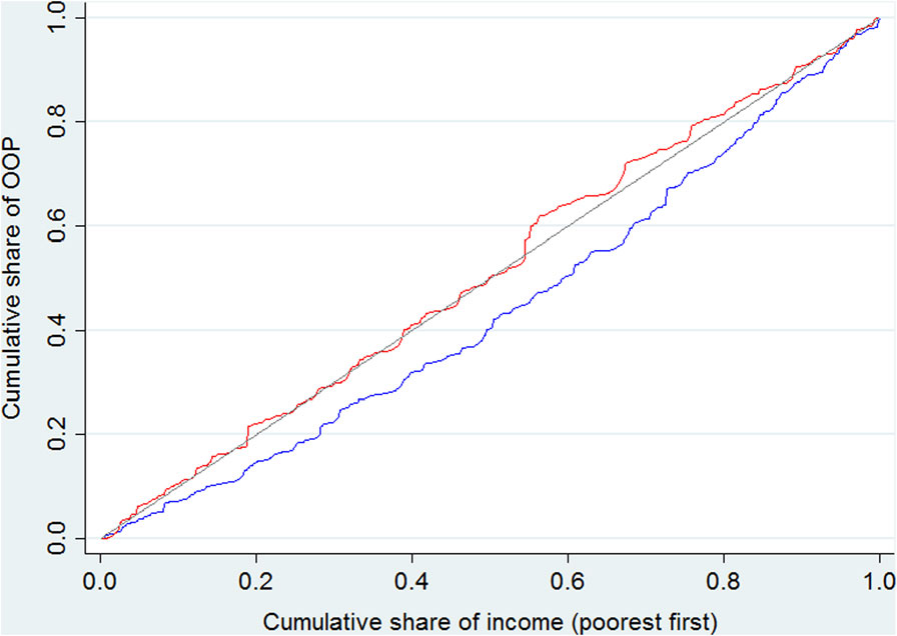
Concentration curve of out-of-pocket health expenditure before and after intervention.  OOP: out-of-pocket

The overall degree of inequality in OOP payments also increased, as is reflected by the positive value of the concentration index before and negative value after implementing the new model (Table 3). This result remains unchanged after adjusting for age, gender and patient category (new or relapse patients). The concentration curves are depicted in Fig. 2, which clearly shows that the accumulated share of total OOP health expenditure paid by poor patients was higher after the intervention, as the curve for the intervention period always lies above the baseline.

**Table 3:** Concentration index of out-of-pocket health expenditure before and after intervention

Period	Crude	Standardized
Before intervention	0.0918	0.0878
After intervention	−0.0262	−0.0049

**Table 4:** Percent of households incurring CHE before and after intervention by income group

Income group	CHE_10/%			CHE_40/%		
	Before intervention	After intervention	*P*-value	Before intervention	After intervention	*P*-value
< 4369 RMB	72.8	82.3	0.016	43.0	52.0	0.055
4369–12 647 RMB	41.6	52.2	0.009	27.3	32.5	0.163
> 12 647 RMB	25.1	31.0	0.221	27.5	22.8	0.316
Overall	47.8	56.3	0.001	32.4	36.5	0.113

CHE_10: 10% of household income threshold; CHE_40: 40% non-food expenditure threshold

### Financial burden

For the overall sample, the percentage of patients that incurred CHE increased from 47.8 to 56.3% using the 10% of household income threshold (CHE_10), and from 32.4 to 36.5% using the 40% non-food expenditure threshold (CHE_40) after implementation of the new model, and the increase was statistically significant for the former (Table 4). The financial burden of TB treatment increased for both lower income groups (*P* <  0.05 for CHE_10). Only patients who were relatively well-off experienced no significant change in either CHE index. It is notable that the financial burden was extremely high for the poorest patients, with over 82.5% of those who lived below the poverty line spending more than 10% of their household income, and a majority spending 40% of their non-food expenditure, on medical services after the intervention.

### Factors associated with the financial burden of TB care

Table 5 shows the results of an OLS regression of the logarithm of direct OOP health expenditure (model 1) and logit regressions of CHE (model 2 and 3) on project intervention, after adjusting for the covariates gender, age, education, employment status, insurance cover and controlling for county fixed effects. Results from the OLS regression again clearly show that implementation of the new model was associated with a significant increase in OOP health expenditure. Results from the logit regressions also reveal a significant increase in the percentage of patients incurring CHE after project implementation for both the 10% of household income and 40% of non-food expenditure thresholds.

**Table 5:** Regression of the logarithm of out-of-pocket and logit regressions of catastrophic health expenditure on an intervention dummy variable and other cofactors

	Model 1: log OOP		Model 2: CHE_10%		Model3: CHE_40%	
	Coef.	*P* > |t|	Odds ratio	*P* > |t|	Odds ratio	*P* > |t|
Intervention period						
after intervention	0.572	0.013	1.86	0.033	1.50	0.021
Income group						
< 4369 RMB	ref.		ref.		ref.	
4369–12 647 RMB	0.217	0.063	0.29	< 0.001	0.59	0.001
> 12 647 RMB	0.436	0.025	0.14	< 0.001	0.72	0.275
Period*income group						
< 4369 RMB	ref.		ref.		ref.	
4369–12 647 RMB	−0.144	0.280	0.81	0.451	0.87	0.286
> 12 647 RMB	−0.426	0.046	0.71	0.168	0.52	0.001
Gender						
Male	0.03	0.743	1.12	0.424	1.13	0.382
Age						
30–59	−0.047	0.868	1.13	0.702	1.47	0.374
≥ 60	0.188	0.454	1.61	0.121	2.90	0.022
Patient category						
Relapse	−0.011	0.911	0.97	0.873	0.87	0.275
Marriage						
Married	0.176	0.004	0.94	0.629	1.01	0.928
Education						
No education	ref.		ref.		ref.	
Primary	0.08	0.410	1.13	0.506	1.42	0.087
Secondary	0.256	0.006	1.26	0.141	1.05	0.800
≥ high school	0.312	0.130	1.32	0.287	1.46	0.014
Insurance type						
Other	ref.		ref.		ref.	
UEMBI	−0.492	0.009	0.41	< 0.001	0.42	0.004
NCMS	−0.441	0.005	0.64	0.016	1.06	0.824
Employment						
Working	−0.246	0.022	0.71	0.002	0.70	< 0.001

CHE_10: 10% of household income threshold; CHE_40: 40% non-food expenditure threshold

*UEBMI* Urban Employee Basic Medical Insurance, *NCMS* New Cooperative Medical Schemes. *Coef.* Coefficient, *OOP* Out-of-pocket

Period*income group: the interaction terms of period (after intervention) and three income groups

Differential influences of the model on the OOP health expenditure and financial burden of patients from different income groups are also observed, as is reflected by the statistically significant coefficients for the interaction term of the intervention period and the highest income group for both models 1 and 3. The OOP health expenditure of this group did not increase as much as that for the poorest, and their financial burden remain relatively unchanged. It is also notable that being covered by UEBMI and NCMS is associated with lower OOP health expenditure and reduced likelihood of incurring CHE.

The qualitative data revealed a variety of stakeholder opinions as to the impact of the model, and provided insights into factors contributing to the high OOP health expenditure and financial burden following the intervention. Policy makers (directors of local health and family planning bureau and health insurance office) in the three study sites generally thought that the new financing and payment model would be beneficial for TB patients and could effectively reduce their financial burden. On the other hand, some directors of the insurance office admitted that patients with serious comorbidities had been excluded from the intervention and thus their costs were not subject to the cost limit set by the case-based payment policy.

*“The policies are good … the reimbursement increased to 70% and patients financial burden are relieved … …*” *(Director of Health and family planning bureau, prefecture level)*

*“Many patients have comorbidities. Originally it was stipulated that the hospitals bear the cost exceeding the fixed rate. However, if the patients have serious comorbidities, like chronic obstructive pulmonary disease and hemoptysis, their treatment costs were really high and sometimes they even need to be transferred to Intensive Care Unit..We could not apply case-based payment for these patients.” (Director of health insurance office, prefecture level)*

Focus group discussions with TB patients reinforced the finding that patients still bear a considerable financial burden for treatment, especially those with comorbidities. Many patients said they took liver-protection and other auxiliary drugs in addition to TB medication, even though these drugs were not included in the standardized TB treatment package. These patients could therefore not enjoy the higher reimbursement rates and nor were the costs of the drugs restricted by the case-based payment limit. In addition, the costs for patients who were transferred to higher level hospitals due to their severe condition were also not covered by the new model. In focus groups discussions with physicians it was argued that the standard treatment package was very limited, which meant that a number of the medical services commonly used by the patients were not included, especially for those with comorbidities. Some physicians also conceded that their income levels were correlated with the net revenue of the department, mainly generated from service provision and drug sales.

*“You have to take these liver-protection drugs, and they cannot be reimbursed for us young people (without special insurance).” (TB patient, FGD)*

*“I had hemoptysis and I was transferred to xxx Hospital (prefecture-level TB designated hospital) from the xx hospital (county-level hospital) … … I was hospitalized for over a month. Then one month after the discharging from the hospital my conditions relapsed, and the doctors in xxx Hospital asked me to go for more checks in xxxx Hospital (prefecture-level general hospital) where I spent over 20 000 RMB … …*” *(TB patient, FGD)*

*“The treatment of comorbidities are not included in the new reimbursement policy … Patients’ complications typically happened when they were readmitted into the hospital, and in this situation they could not enjoy the new policy either.” (TB physician, county-level, FGD)*

*(Our income) is correlated with the net income of the department. He (the hospital manager) set a line …*. F*or example, you treat 100 patients and bring in such an income, and how much you can take from the income … (TB physician, prefecture-level, FGD)*

## Discussion

This study shows that the average OOP health expenditure and financial burden on the patients increased after implementing the new financing and payment mode. National statistics show that the average outpatient cost per visit increased 13.3% and average inpatient cost per admission increased 11.1% over the intervention period. Clearly, the percentage increase following the intervention was much higher and cannot be fully explained by the general rising trend in healthcare costs [34]. As evidenced in the companion paper by Dong et al., the new model contributed to a substantially increased use of outpatient and inpatient services, especially for the lowest income group. Their hospitalization rate increased from 48.5 to 70.7%, and the number of outpatient visits from 4.6 to 5.7. From an equity prospective, the reduced differences in service use across different income groups can be seen as a positive outcome of the intervention. No one would wish to see poorer patients avoiding CHE by choosing not to access the services they need. Nevertheless, the intervention also resulted in an increased inequality in OOP payments for TB care. Clearly the model did not provide sufficient financial protection to offset the substantially increased costs resulting partly from the increase access promoted by the intervention, and both the project designers and implementers at the national and local levels may not have fully anticipated this situation.

Qualitative results from the study offer several possible reasons why poorer patients paid more out-of-pocket during the intervention period. While the new model encouraged poor patients to start and adhere to regular treatment, many of the medical services they received were not included in the standard treatment package, and were thus not reimbursed at a higher rate. The costs of these medical services were also not restricted by the case-based payment limit as defined by the project policies. Studies have shown that patients in lower socio-economic status tend to delay treatment [35], and thus they are more likely to result in more treatments for complications or comorbidities eventually, thus increasing out-of-pocket payments for services not covered by the model. Moreover, if their condition was severe, they risked being excluded from the project because physicians in these designated hospitals were concerned that their department/unit would have to incur a deficit if the treatments they provide to the patients with serious co-morbidities are paid for using the case-based payment. Alternatively, they might be transferred to higher level hospitals where they would not enjoy the benefits of the new policies. From the physician side, as their incomes are closely related to the net income of the department, they had an incentive to exclude patients with severe comorbidities from the program, or prescribe medical services outside the treatment package to increase revenue.

In light of the financial difficulty faced by the poor patients, future policies concerning the financing of TB treatment might need to consider a strategy that is more supportive. A systematic review on interventions to reduce illness and injury related financial burden shows that eliminating or largely reducing copayments in current insurance schemes is effective in reducing OOP medical payments [36]. Evidence from cash transfer programs to support TB households in low and middle income countries also shows that this strategy could increase the TB cure rate [37], and one study in Peru also finds a reduction in CHE [38]. Based on this international experience and findings from our study, improving the reimbursement rate for all TB related medical services is necessary to reduce poor TB patients’ financial burden. However, it is also critical to ensure that the costs of all these medical services are restricted by the case-based payment; otherwise there remains an incentive for physicians to pursue income by prescribing unnecessary drugs and treatments. In addition, more financial resources, for example earmarked funds from government, should be mobilized to subsidize poor patients, especially those with complications and comorbidities.

This study has several limitations. One major limitation is that we did not have a formal list of patients who were included in the program throughout their treatment and those who were excluded. Nevertheless, as the new model was required by the government to be implemented in all designated hospitals, it would seem likely that the vast majority of patients were included. It can also be argued that assessment of the model under the current health system implies that its effectiveness may have been influenced by implementation barriers known to be imbedded in that system, for example the tendency for hospitals to avoid innovations that they perceive as potential threats to income generation. We did not ask about complications and comorbidities in the survey, and we cannot exclude the possibility that the patients enrolled in treatment during the project had more severe conditions and incurred higher costs for this reason. In addition, recall bias is likely as we asked patients about the whole treatment period since the onset of symptoms.

## Conclusions

The mean out-of-pocket health expenditure and financial burden of TB patients increased after the implementation of the new model and we would argue that the limitations mentioned above did not substantially influence this conclusion, given the degree of the observed increase. Apparently, the new financing and payment model was not successful in protecting poor patients from financial difficulty during their treatment. Future research may be required to look into strategies to provide more financial protection for poorer patients and impose effective cost control.

## Additional file

Additional file 1: Multilingual abstracts in the five official working languages of the United Nations. (PDF 563 kb)

## Abbreviations

CHE: Catastrophic health expenditure
DRG: Diagnostic-related group
FGD: Focus-group discussion
NCMS: New cooperative medical schemes
OOP: Out-of-pocket
TB: Tuberculosis
UEBMI: Urban employee basic medical insurance
URBMI: Urban resident basic medical insurance
