# Supplementary material for: Impact of an innovative financing and payment model on tuberculosis patients’ financial burden: is tuberculosis care more affordable for the poor?
Source: Infect Dis Poverty. 2019 Mar 24;8:21. doi: 10.1186/s40249-019-0532-x (PMC6431427; doi:10.1186/s40249-019-0532-x)
Supplement: Supplementary file 1 — Multilingual abstracts in the five official working languages of the United Nations. (PDF 563 kb) [file 40249_2019_532_MOESM1_ESM.pdf]

Translation of the abstract into the five official working languages of the United Nations

أثر نموذج مبتكر للتمويل والدفع على العبء المالي لمرضى السل: هل رعاية مرضى السل ميسورة التكلفة للفقراء؟

Chen, Li Xiang, Qiang Li, Fei Huang, Hong Ying-Xi Jiang, Qian Long, Henry Lucas, Di Dong, Jia-Wei
Lan Tang-Wang, Chris Elbers, Frank Cobelens, Sheng

نُبذة

خلفيةاستجابة للعبء المالي المرتفع للخدمات الصحية التي تواجه مرضى السل في الصين ، قام مشروع الصين-غيتس للسل ، المرحلة الثانية ، بتنفيذ نموذج جديد للدفع والتمويل كمكون مهم للمشروع الإجمالي في ثلاث مدن في شرق البلاد. وسط وغرب الصين. ويركز النموذج على زيادة معدل السداد لمرضى السل وإصلاح طرق سداد المورّدين عن طريق استبدال الرسوم مقابل الخدمة بنهج الدفع القائم على الحالة. بحثت هذه الدراسة في التغيرات في الدفع المباشر (OOP) لنفقات الصحة والعبء المالي على مرضى السل قبل وبعد التدخلات ، مع التركيز على التأثيرات المختلفة المحتملة على المرضى من مجموعات الدخل المختلفة.

الأساليب: ثلاث مقاطعات عينة في كل من المحافظات الثلاث: تم اختيار تشنجيانغ وييتشانغ وهانتشونغ كمواقع للدراسة. مرضى السل الذين بدأوا وأكملوا العلاج من قبل ، وخلال فترة التدخل ،تم أخذ عينات منهم بشكل عشوائي وتم فحصهم في الأساس في عام 2013 والتقييم النهائي في عام 2015 على التوالي. تم احتساب نفقات الصحة ونسبة المرضى الذين يعانون من نفقات صحية ضخمة (ECH) لفئات الدخل المختلفة. تم إجراء انحدار OLS والانحدار اللوجستي لاستكشاف آثار التدخل على المريض الأنفاق المباشر علي النفقات الصحية والعبء المالي بعد التعديل للمتغيرات الأخرى. أجريت المقابلات الرئيسية ومناقشات مجموعة التعليقات لفهم أسباب أي تغييرات تم ملاحظتها.

النتائج :البيانات من 738 (خط الأساس) و 735 (التقييم) المرضى كانوا متاحيين للتحليل. زيادة متوسط المرضى الإنفاق الصحي على الدفع المباشر من 3،576 يوان إلى 5،791 يوان ، كما زادت النسبة المئوية للمرضى الذين يتكبدون نفقات صحية ضخمة بعد التدخل. كانت النسبة المئوية للزيادة في الأنفاق المباشر للنفقات الصحة واحتمالية تكبد نفقات صحية ضخمة أقل بشكل ملحوظ بالنسبة للمرضى من أعلى مجموعة دخل بالمقارنة مع الأقل. أشارت النتائج النوعية إلى أن زيادة استخدام الخدمات الصحية التي لا تغطيها الحزمة الاساسية للنموذج من المرجح قد تكون سببت زيادة العبء المالي.

الاستنتاج:إن تنفيذ نموذج التمويل والمدفوعات الجديد لا يحمي المرضى ، من الصعوبات المالية ، ولا سيما أولئك الذين ينتمون إلى مجموعة الدخل الأدنى ، ويرجع ذلك جزئيا إلى زيادة استخدامهم للخدمات الصحية. يجب جمع المزيد من الموارد المالية لزيادة الحماية المالية ، وخاصة للمرضى الفقراء ، في حين يجب وضع استراتيجيات لاحتواء التكاليف وتنفيذها بفعالية لتحسين التغطية الفعالة للرعاية الصحية الأساسية في الصين.

Translated from English version into Arabic by Mohamed Shawkat, proofread by Aalya Al-Beeshi, through

TRANSLATORS
WITHOUT BORDERS

创新筹资支付模型对结核患者经济负担的影响：结核医疗服务对贫困患者而言是否更可负担？

Wei-Xi Jiang, Qian Long, Henry Lucas, Di Dong, Jia-Ying Chen, Li Xiang, Qiang Li, Fei Huang, Hong Wang, Chris Elbers, Frank Cobelens, Sheng-Lan Tang

摘要

引言：为应对中国结核患者使用医疗服务时面临的高经济负担，中国-盖茨基金会结核病项目二期在中国东、中和西部的三个城市实施了新型筹资支付模型，作为整个项目的重要组成部分。新

模型致力于提高结核患者的医保报销比例，并以按病种付费的支付方式代替原有的按项目付费。本研究调查了干预模型实施前后结核患者自付费用和经济负担的变化，并特别关注模型对于不同收入水平患者潜在的不同影响。
方法：项目在三个地级市：镇江、宜昌和汉中各选取了三个样本县作为研究现场，在 2013 年的基线调查中随机抽取干预实施前就开始并完成治疗的患者，在 2015 年的终末评估中随机抽样调查在干预期间内开始并完成治疗的患者。本研究对不同收入的人群分别计算了自付医疗费用和发生灾难性支出的比例，并应用普通最小二乘法回归和逻辑回归分析，在校正协变量的基础上探究干预对于患者自付医疗费用和经济负担的影响。本研究还进行了关键人物访谈和小组访谈，以了解费用和经济负担产生变化的原因。
结果：基线和终末分别有 738 和 735 例患者样本可供分析。患者人均自付医疗费用从 3576 人民币上涨到 5791 人民币，发生灾难性支出的患者比例也在干预后有所上升。最富裕的患者自付医疗费用和发生灾难性支出的概率增长的百分比显著低于最贫困的患者。定性研究的结果表明标准临床服务包外的医疗服务利用增长，而这部分费用无法被新筹资支付模型覆盖，可能导致患者经济负担增加。
结论：项目实施的新型筹资支付模型未能保护最贫困的患者人群免于经济困难，其中部分原因是医疗服务利用的增加。因此应该多渠道增加筹资，以增加对贫困患者的经济保护，此外费用控制的额策略也亟待开发和实施，以提升中国的基本医疗卫生服务的有效覆盖。

Translated from English version into Chinese by Wei-Xi Jiang

Impact d’un modèle innovant de financement et de rémunération sur le poids financier de la tuberculose pour les patients : le traitement de la tuberculose est-il plus abordable pour les plus démunis?

Wei-Xi Jiang, Qian Long, Henry Lucas, Di Dong, Jia-Ying Chen, Li Xiang, Qiang Li, Fei Huang, Hong Wang, Chris Elbers, Frank Cobelens, Sheng-Lan Tang

Résumé

Contexte: face au poids financier important que représente le traitement de la tuberculose pour les patients en Chine, la Phase II du projet China-Gates TB a déployé un nouveau modèle de financement et de rémunération, composante importante d’un projet plus large, dans trois villes de l’est, du centre et de l’ouest de la Chine. Ce modèle prévoit l’augmentation du taux de remboursement des patients tuberculeux et la réforme des méthodes de rémunération des prestataires, remplaçant le paiement à l’acte par une approche au cas par cas. L’étude a évalué l’évolution des avances de frais et le poids financier supporté par les patients tuberculeux avant et après les interventions, en se concentrant sur les possibles différences d’impact au sein de différents groupes de revenus.
Méthodes: trois circonscriptions pilotes ont été choisies dans les trois préfectures : Zhenjiang, Yichang et Hanzhong. Les patients tuberculeux ayant commencé et terminé un traitement avant et pendant la période d’intervention ont été randomisés et évalués au début de l’étude, en 2013, et à la fin en 2015. Les avances de frais médicaux et le pourcentage de patients supportant des frais médicaux extrêmement élevés ont été calculés pour différents groupes de revenus. Une régression linéaire et une régression logistique ont été effectuées afin d’explorer l’impact de l’intervention sur les avances de frais médicaux des patients et le fardeau financier, après ajustement en fonction d’autres covariables. Des entretiens avec des informateurs clés et des discussions en groupes thématiques ont été menés afin de comprendre les

raisons des changements observés.
Résultats: les données de 738 patients au début de l’étude et 735 lors de l’évaluation étaient disponibles pour l’analyse. Les avances de frais médicaux moyennes par patient ont augmenté de 3576 RMB à 5791 RMB et le pourcentage de patients ayant supporté des frais médicaux extrêmement élevés était également supérieur après l’intervention. Le pourcentage d’augmentation des avances de frais et la probabilité de frais médicaux extrêmement élevés étaient significativement plus faibles au sein du groupe des revenus les plus élevés par rapport à celui des revenus les plus bas. Les observations qualitatives suggèrent que cette augmentation du fardeau financier résulte du recours accru à des services de santé qui n’étaient pas couverts par le plan standard du modèle.
Conclusions: la mise en place d’un nouveau modèle de financement et de rémunération n’a pas protégé les patients, notamment ceux du groupe aux revenus les plus faibles, contre les difficultés financières, en partie à cause d’un plus important recours aux services de santé. Il faudrait mobiliser davantage de ressources financières pour accroître la protection financière, en particulier celle des patients les plus démunis, tout en mettant au point des stratégies de maîtrise des coûts et en les déployant efficacement afin d’améliorer la couverture par les soins de santé essentiels en Chine.

Translated from English version into French by Suzanne Assenat, proofread by Eric Ragu, through

TRANSLATORS
WITHOUT BORDERS

Инновационные инструменты финансирования и оплаты медицинских услуг и финансовое бремя больных туберкулезом: становится ли лечение туберкулеза более доступным для бедняков?

Вэй-Си Цзян, Цянь Лун, Генри Лукас, Ди Дун, Цзя-Ин Чэнь, Ли Сянь, Цян Ли, Фэй Хуан, Хун Ван, Крис Элберс, Франк Кобеленс, Шэн-Лань Тан

Аннотация

Справочная информация. В Китае лечение туберкулеза (ТБЦ) оборачивается для пациентов значительным финансовым бременем. Чтобы изменить ситуацию, в рамках второго этапа осуществления проекта China-Gates по борьбе с ТБЦ была реализована новая модель финансирования и оплаты медицинских услуг; она стала важным элементом проекта, который осуществляется в трех крупных городах в восточной, центральной и западной частях страны. В рамках новой модели повышается доля расходов на лечение, возмещаемых больным ТБЦ, и изменяется система формирования оплаты медицинских услуг: вместо оплаты услуг по отдельности, пациенты оплачивают лечение единым пакетом. В настоящей работе анализируется изменение затрат из собственных средств (ЗСС) пациентов на оплату медицинских услуг и общего финансового бремени лечения ТБЦ до и после внедрения новой модели. Основное внимание уделяется последствиям внедрения новой модели для групп населения с различными уровнями доходов.
Методика. Для проведения анализа были выбраны три района, входящие в префектуры Чжэньцзян, Ичан и Ханьчжун. В рандомизированную выборку были включены пациенты,

начавшие и завершившие лечение ТБЦ до и в течение периода введения в действие новой модели. За базовый уровень были приняты показатели 2013 года, для конечной оценки были взяты показатели 2015 года. Для отдельных групп, сформированных по уровням доходов, оценивались ЗСС на оплату медицинских услуг, после чего определялось процентная доля пациентов, понесших катастрофически высокие затраты (КВЗ). По результатам МНК-регрессии и логистической регрессии было определено воздействие новой модели на ЗСС пациентов на медицинские услуги и, после корректировки с учётом других предикторов, на размер финансового бремени. Для понимания причин выявленных изменений были проведены интервью с ключевыми информантами и обсуждения в фокус-группах.

Результаты. В рамках анализа оцениваемые показатели (735 пациентов) сравнивались с базовыми показателями (738 пациентов). Средний размер ЗСС пациентов на оплату медицинских услуг увеличился с 3576 юаней до 5791 юаней; процентная доля пациентов, понесших КВЗ, также увеличилась. В сравнении с наименее обеспеченной группой, для пациентов из наиболее обеспеченной группы процентный рост ЗСС был значительно ниже, как и риск понести КВЗ. Результаты качественного характера свидетельствуют, что увеличение объема потребляемых услуг здравоохранения, стоимость которых не покрывается за счет предусмотренного новой моделью стандартного пакета, с большой вероятностью ведет к увеличению финансового бремени.

Выводы. Внедрение новой модели финансирования и оплаты не обеспечило пациентам должную защиту от финансовых затруднений, частично – ввиду увеличения потребления медицинских услуг; в первую очередь это испытали на себе представители наименее обеспеченных групп. Для более надежной финансовой защиты необходимо мобилизовать дополнительные финансовые ресурсы. Кроме того, необходимо обеспечить разработку и эффективную реализацию стратегий сдерживания затрат, что позволит расширить охват населения Китая базовыми медицинскими услугами.

Translated from English version into Russian by Alexander Poddubnyy, proofread by Tatiana Fridman, through

TRANSLATORS
WITHOUT BORDERS

Impacto de un modelo innovador para la financiación y el pago de los gastos de los pacientes con tuberculosis: ¿se logra una atención sanitaria más asequible para los pobres?

Wei-Xi Jiang, Qian Long, Henry Lucas, Di Dong, Jia-Ying Chen, Li Xiang, Qiang Li, Fei Huang, Hong Wang, Chris Elbers, Frank Cobelens, Sheng-Lan Tang

Resumen

Contexto: como respuesta a la elevada carga financiera a que se enfrentan los pacientes con tuberculosis (TB) para acceder a la atención sanitaria en China, el proyecto de TB China-Gates, Fase II, ha implementado un nuevo modelo de financiación y pago como componente importante del proyecto global en tres ciudades del este, centro y oeste de China. El modelo se centra en aumentar la tasa de

reembolso para pacientes con tuberculosis y en reformar los métodos de pago de los proveedores mediante el reemplazo de la tarifa por servicio por un esquema de pago basado en los casos. Este estudio investigó los cambios en los gastos sanitarios de bolsillo y la carga financiera en pacientes con TB antes y después de las intervenciones, centrándose en los posibles impactos diferenciales en pacientes de diferentes grupos de ingresos.

Métodos: muestra de tres municipios en cada una de las tres prefecturas: se eligieron Zhenjiang, Yichang y Hanzhong como lugares de estudio. Se encuestó a los pacientes con TB que habían iniciado y completado el tratamiento antes y durante el período de intervención y se tomaron muestras de manera aleatoria al comienzo en 2013 y en el momento de la evaluación final en 2015, respectivamente. Los gastos sanitarios de bolsillo y el porcentaje de pacientes que incurrieron en gastos sanitarios catastróficos se calcularon para los diferentes grupos de ingresos. La regresión MCO y la regresión logit se realizaron para explorar los efectos de la intervención en el gasto sanitario de bolsillo y la carga financiera de los pacientes después de ajustar otras covariables. Se realizaron entrevistas con informantes clave y debates de grupos focales para comprender las razones de los cambios observados.

Resultados: para el análisis estuvieron disponibles los datos de 738 (comienzo) y 735 (evaluación) pacientes. El gasto sanitario de bolsillo medio de los pacientes aumentó de 3.576 RMB a 5.791 RMB y el porcentaje de pacientes que incurrieron en gastos sanitarios catastróficos también aumentó después de la intervención. El porcentaje de aumento del gasto sanitario de bolsillo y la probabilidad de incurrir en gastos sanitarios catastróficos fueron significativamente más bajos para los pacientes del grupo de mayores ingresos en comparación con el de menores ingresos. Los hallazgos cualitativos indicaron que el aumento en el uso de los servicios de salud no cubiertos por el paquete estándar del modelo probablemente haya ocasionado el aumento de la carga financiera.

Conclusiones: la implementación del nuevo modelo de financiación y pago no protegió a los pacientes, especialmente a los del grupo de menores ingresos, de las dificultades financieras, debido en parte a su mayor uso del servicio de salud. Se deben movilizar más recursos financieros para aumentar la protección económica, especialmente para los pacientes pobres, mientras que las estrategias de contención de gastos deben desarrollarse e implementarse eficazmente para mejorar la cobertura efectiva de la atención médica esencial en China.

Translated from English version into Spanish by Raquel Osuna García, proofread by Mayra León, through

TRANSLATORS WITHOUT BORDERS
